# Phosphorus Nutrition in Songpu Mirror Carp (*Cyprinus carpio* Songpu) During Chronic Carbonate Alkalinity Stress: Effects on Growth, Intestinal Immunity, Physical Barrier Function, and Intestinal Microflora

**DOI:** 10.3389/fimmu.2022.900793

**Published:** 2022-06-29

**Authors:** Ze Fan, Di Wu, Jinnan Li, Chenhui Li, Xianhu Zheng, Liansheng Wang

**Affiliations:** Key Laboratory of Aquatic Animal Diseases and Immune Technology of Heilongjiang Province, Heilongjiang River Fisheries Research Institute, Chinese Academy of Fishery Sciences, Harbin, China

**Keywords:** carbonate alkalinity, phosphorus, antioxidant capacity, intestinal microflora, immunity, Songpu mirror carp (*Cyprinus carpio* Songpu)

## Abstract

Carbonate alkalinity is a major environmental stress factor affecting aquatic feed configuration, which easily causes oxidative stress and hypoimmunity for fish. Hence, the purpose of the study is to assess the potential effect of phosphorus on growth, intestinal oxidation resistance, physical barrier function, and microflora for Songpu mirror carp (*Cyprinus carpio* Songpu) (initial average weight of 2.95 ± 0.21 g) reared at the high-concentration carbonate alkalinity environment. A two-factor, three-level (2 × 3) design was applied, in which diets with three different phosphorus levels (3.6, 7.0, and 10.5 g/kg dry matter) were randomly assigned to 0 and 15 mmol/L carbonate alkalinity groups with three replicate aquariums. After the 8-week trial, we found that weight gain rate (WGR), specific growth rate (SGR), protein efficiency ratio (PER), and lipase and amylase activities in the intestine significantly (*p* < 0.05) declined with increasing carbonate alkalinity. Carbonate alkalinity of 15 mmol/L significantly reduced glutathione peroxidase (GSHPx) activities in the intestine (*p* < 0.05). The relative expressions of nuclear factor (erythroid-derived 2)-like 2 (Nrf2), glutathione peroxidase 1a (GPX1a), Clautin3, Clautin11, and tumor necrosis factor β (TNF-β) in the intestine were markedly downregulated by increasing carbonate alkalinity levels (*p* < 0.05), whilst the relative expressions of interleukin 1β (IL-1β) and tumor necrosis factor α (TNF-α) in the intestine were markedly upregulated (*p* < 0.05). At the 15 mmol/L carbonate alkalinity treatment, Songpu mirror carp suffer from hypoimmunity status with failed digestion, antioxidant, inflammation, and immune response, thereby inducing impaired growth. Additionally, significant increments in the abundance of Proteobacteria and a significant decrease in the abundance of Fusobacteria and the Firmicutes/Bacteroidetes ratio were caused due to excessively high carbonate alkalinity (15 mmol/L) and excessively low dietary phosphorus supply (3.6 g/kg). Collectively, 7.0 g/kg dietary phosphorus supplementation was effective in promoting intestinal antioxidant enzyme activities and the corresponding gene expression *via* the Keap1-Nrf2 signaling pathway and in enhancing intestinal immunity by upregulating anti-inflammatory and downregulating pro-inflammatory genes. Appropriate dietary phosphorus supply could promote the formation of beneficial microflora in freshwater, and it has the potential ability to transfer the adverse effect of carbonate alkalinity stress to the structural composition of intestinal microflora. Hence, consideration should be given to suitable phosphorus supply for fish under the chronic carbonate alkalinity stress.

## Introduction

Various environmental factors are the crucial elements influencing the growth of fish. In general, the culture environment exerts influence on nutritional requirements to some extent. Carbonate alkalinity is a relatively major environmental stress factor, especially in northeast China and northwest China (1). Long-term carbonate alkalinity stress not only can impair the growth, immunity, and disease resistance of aquatic animals but also can have an impact on nutrient metabolism *in vivo* for fish (2, 3). For instance, because chronic carbonate alkalinity stress of 15 and 30 mmol/L can conspicuously suppress growth, the antioxidant capacity and ammonia metabolism of Songpu mirror carp (*Cyprinus carpio* Songpu) were limited during the 8-week breeding process, whereas 10 g/kg addition of AKG can reverse that disadvantages induced by chronic carbonate alkalinity stress (4). A recent study from our laboratory indicated that a dietary protein supply of 310 g/kg could effectively limit the oxidative damage and strengthen the intestinal immune response of fish to cope better with the long-term carbonate alkalinity stress of 15 mmol/L (5). These data suggested that the link between carbonate alkalinity and fish nutritional requirements needs to be evaluated scientifically.

Phosphorus intake has been deemed as one of the most critical elements in promoting growth and maintaining metabolic balance (6). However, by reason of the low-concentration soluble inorganic phosphorus and phosphate, which cannot meet the demand of fish, suitable and sufficient feed supply has become the dominating source of phosphorus for fish (7, 8). From the perspective of deficiency in dietary phosphorus, growth would be suppressed by the skeletal abnormality (9), low feed efficiency (10), weak digestion ability (11), infirm immunity, and antioxidant capacity (12), whilst from the perspective of excess dietary phosphorus, increasing phosphorus excretion into the water can cause culture water pollution. Although many studies have involved dietary phosphorus requirements under the various environmental factors such as temperature (13), light (13), stocking density (14), and salinity (15), the comprehensive influence of phosphorus nutrition and carbonate alkalinity has not been previously assessed.

The changes in the immune indices in the intestine can potentially affect the health state of fish (16). Related immune indices mainly cover antioxidant ability, cytokines, epithelial cells, tight junction (TJ) complex, intestinal microflora, etc., which play crucial roles in nutrient absorption and host defense to a hostile environment and poor nutritional conditions for fish (17, 18). Although it has been reported that the interplay between carbonate alkalinity and α-ketoglutarate or protein can improve the partial immune indices (4, 5), so far, such report on the interaction on the immune indices between carbonate alkalinity and phosphorus has still remained unknown. Based on these, the study aimed to make an exploration analysis on the underlying interaction between dietary phosphorus supply and carbonate alkalinity, basically concerning intestinal immunity and microflora by taking Songpu mirror carp as the study object, which has been widely cultivated throughout most areas of China and has the obvious superiority in survival rate, disease resistance, and anti-stress ability (19). Thus, we determined the growth, intestinal immunity, physical barrier function, and intestinal microflora in Songpu mirror carp fed with different diets containing different dietary phosphorus levels and maintained at different carbonate alkalinity. The findings will be compared to illustrate the effects of high-concentration carbonate alkalinity on Songpu mirror carp phosphorus requirement to provide scientific guidance for helping Songpu mirror carp resist carbonate alkalinity stress in its local aquatic environment.

## Materials and Methods

### Experimental Diet Formulation and Preparation

According to National Research Council (NRC) (2011) (20) guidelines, three isoproteic (301 g/kg) experimental diets with three different phosphorus levels were designed (**Table 1**). Monosodium phosphate (NaH_2_PO_3_) served as the main phosphorus source and was supplemented to attain the expected levels (low phosphorus level (LP), 3.6 g/kg dry matter; normal phosphorus level (NP), 7.0 g/kg dry matter; and high phosphorus level (HP), 10.5 g/kg dry matter) in line with the study on Jian carp (*C. carpio* var. Jian) of Dong et al. (21). In order to accomplish the different levels of dietary phosphorus in the experiment diets, the additive amount of monosodium phosphate was elevated with the decrease of dietary cellulose. The rough material was adequately smashed and sifted through a 60-mesh sieve. Vitamin premix, mineral premix (phosphorus free), l-methionine, l-threonine, choline chloride, and chromium sesquioxide (Cr_2_O_3_), etc., which has an additive amount of no more than 10 g/kg as shown in **Table 1**, were blended first and then thoroughly intermingled with the other ingredients. Before pelleting, fish oil, soybean oil, phospholipids, and distilled water were later added until a smooth dough was obtained. All the diets were granulated into 1.5-mm pellets by a laboratory pellet presser (GYJ-250B, Dashiqiao Bao’s Feed Machinery Factory, Harbin, China). The obtained pellets were air-dried and kept at −20°C till use.

**Table 1:** Formulation and nutrritional proximate compositions of the experimental diets (g/kg dry matter).

Ingredients	Phosphorus levels		
	LP	NP	HP
	3.6	7.0	10.5
Soybean protein concentrate	250	250	250
Corn starch	382	382	382
Gelatin	60	60	60
Casein	80	80	80
Fishmeal	30	30	30
Wheat middling	60	60	60
Fish oil	35	35	35
Soybean oil	10	10	10
Phospholipid	20	20	20
Sodium carboxymethylcellulose	20	20	20
Vitamin premix^a^	3	3	3
Trace mineral premix (Phosphorus free)^b^	2	2	2
L-methionine	2	2	2
L-threonine	2	2	2
Choline chloride	4	4	4
Monosodium phosphate (NaH_2_PO_3_)	7	21	35
Cellulose	28	14	0
Chromium sesquioxide (Cr_2_O_3_)	5	5	5
Proximate composition			
Crude protein	301.1	301.5	301.8
Crude lipid	71.9	71.4	71.8
Crude ash	24.5	24.9	24.1
AvailABLE phosphorus	2.9	4.9	7.1

a Vitamin mixture (g/kg mixture) supplied by Guangdong Hyint Biotechnology Group Co. Ltd: vitamin A(VA) 8,000 IU, vitamin C (VC) 100 mg, vitamin D_3_ (VD_3_) 3,000 IU, vitamin E (VE) 60 mg, vitamin K_3_ (VK_3_) 5 mg, vitamin B_1_ (VB_1_) 15 mg, vitamin B_2_ (VB_2_) 30 mg, vitamin B_6_ (VB_6_ ) 15 mg, vitamin B_12_ (VB_12_) 0.5 mg;

b Trace mineral mixture (mg/g mixture) supplied by Guangdong Hyint Biotechnology Group Co. Ltd: nicotinamide 175 mg, d-biotin 2.5 mg, inositol 1,000 mg, folic acid 5 mg, pantothenic acid 50 mg, zinc (Zn) 60 mg, copper (Cu) 3 mg, iron (Fe) 25 mg, manganese (Mn) 15 mg, iodine (I) 0.6 mg, and magnesium (Mg) 0.7 mg.

### Feeding Management

A two-factor, three-level (2 × 3) design was applied, with three different phosphorus levels and two carbonate alkalinity (C0, 0 mmol/L; and C15, 15 mmol/L) according to our previous study (4, 5).

Analytically pure sodium bicarbonate (the product purity ≥99.5%, Tianjin Zhiyuan Chemical Reagent Co., Ltd., Tianjin, China) was configured as the corresponding carbonate alkalinity, and 0.02 mmol/L of HCl was applied to correct it. Mixed methyl orange-aniline blue and phenolphthalein were chosen as indicators, and the measured carbonate alkalinity and pH are detailed in **Table 2**. Throughout the 8-week experiment, the water temperature was adjusted to 22°C–24°C. The water dissolved oxygen was beyond 5 mg/L, and 30% of water was exchanged every couple of days to ensure good water quality. Meanwhile, the carbonate alkalinity of water was adjusted to the unified concentration using the pre-configured mother liquor throughout the entire trial. All water quality indices were detected with a multiparameter water quality analyzer (HQ40D) (HACH Company, Loveland, CO, USA).

**Table 2:** Designed and measured carbonate alkalinity N=6; means±SD; mmol/L.

Caobonate alkalinity and pH	Groups	
	0 mmol/L	15 mmol/L
Disigned caobonate alkalinity	0.00	15.0
Measured caobonate alkalinity	0.00	14.9±0.1
pH	6.95±0.16	8.27±0.16

The feeding management was the same as in our previous research (5). Healthy and similarly sized juvenile Songpu mirror carp were acquired from the breeding farm of Hulan Experimental Station of Heilongjiang Fisheries Research Institute (Harbin, China). The carp were acclimatized for 2 weeks and fed with a commercial diet (Tong Wei Co. Ltd., Chengdu, China; protein content, 30%) three times a day. The trial was carried out in an indoor aquarium with a recirculating aquaculture system (a water volume of 200 L) and an auto-supplemented oxygen system. To acclimate to the experimental conditions, they were raised in several aquariums (200 L) to acclimate to the experimental conditions and fed with a commercial diet containing 320 g/kg of crude protein (Tong Wei CO. Ltd.) three times a day. In total, 450 fish (initial average weight of 2.95 ± 0.21 g) from the acclimatization aquarium were randomly allotted into 18 experimental aquariums (triplicate aquariums). The fish were hand-fed to apparent satiety three times daily at 08:00, 13:00, and 17:00 for 8 weeks.

### Sample Collection and Analysis

Based on the experimental procedure of the Association of Official Agricultural Chemists (AOAC) (2006) (22), the composition analysis of diets was carried out. At the end of the trial, two fish in each aquarium were sampled to obtain the intestine samples. The intestine samples were divided into two sections. One section was frozen at −20°C until enzyme activity testing. The detection details of the composition of diets and the intestinal activities of digestion, antioxidant factors, and immune-related enzymes are presented in **Table 3**, as in our previous research.

**Table 3:** The chemical analysis used in the experiment.

Items	Methods (NO.)	Reference/Assay kits
**Composition of diets**		
Moisture	Drying at 105 °C to constant weight	Association of Official Analytical Chemists (AOAC) (2006) (22)
Crude protein	Kjeldahl method	
Crude lipid	Ether extraction method with a Soxtec system	
Crude ash	Combustion to a constant weight at 550°C	
**Intestinal activities of antioxidant factors and immune-related enzymes**		
Superoxide dismutase (SOD)	WST-1 method (A001-3-2)	Assay kits purchased from Jian Cheng Bioengineering Institute (Nanjing, China)
Malondialdehyde (MDA)	Thiobarbituric acid (TBA) method (A003-1-2)	
Glutathione peroxidase (GSH-Px)	Ultraviolet colorimetry (A005-1-2)	
Complement 3 (C3)	Immunoturbidimetry (E032-1-1)	
Complement 4 (C4)	Immunoturbidimetry (E033-1-1)	
Alkaline phosphatase (AKP)	Microplate method (A059-2-2 )	
Acid phosphatase (ACP)	Microplate method (A060-2-2 )	

### Analysis of Gene Expression

The other section was frozen at −80°C for later analysis of gene expression.

#### RNA Extraction

Total RNA in the intestine samples was isolated referring to the manufacturer’s recommendations of RNAiso Plus. The purity and quantitative ratios of absorbance at 260 and 280 nm respectively were quantified by spectrophotometry and controlled near 2.0. The integrity of the RNA was determined by 1.0% formaldehyde denaturing agarose gels.

#### cDNA Synthesis

First, the concentration of RNA samples was adjusted to 1,000 ng/μl. Inverse transcription was conducted using TaKaRa PrimeScript™ RT reagent Kit with gDNA Eraser (Perfect Real Time) (Code No. RR047A) (Dalian Takara Company, Dalian, China).

#### Quantitative RT-PCR

According to the instructions of the TaKaRa SYBR^®^ Premix Ex Taq™ (Tli RNaseH Plus) (Code No. RR420A) (Dalian Takara Company), the specific reaction mixture was performed through a 20-μl reaction mixture including SYBR^®^ Premix DimerEraser (10 μl), PCR forward primer (0.4 μl), PCR Reverse Primer (0.4 μl), cDNA template (≈100 ng, 2 μl) (**Table 4**), and RNase-free dH_2_O (7 μl). The primers in **Table 4** were gathered from the obtained sequences of the National Center for Biotechnology Information (NCBI), and β-actin was deemed as an internal reference gene, the amplification efficiency of which was calculated depending on the specific gene standard curves and generated from 10-fold serial dilutions. Quantitative RT-PCR was executed with ABI 7500 real-time PCR machine (ABI, Applied Biosystems, Foster City, CA, USA). The relative expression levels of each gene were calculated depending on the comparative CT method (2^−ΔΔCt^) method (23).

**Table 4:** Primers used for quantitative RT-PCR (qPCR).

Gene name	Primer sequence (5′-3′)	Gene serial number
Nfr2^1^	F: TTCCCGCTGGTTTACCTTAC R: CGTTTCTTCTGCTTGTCTTT	JX462955
Keap1^2^	F: CTACAACCCCGAGAGACGA R: GGAGGAGATGAAGCTCCAGAC	JX470752
CuMnSOD^3^	F:TGGCGAAGAAGGCTGTTTGT R:TTCACTGGAGACCCGTCACT	JF342355
CAT^4^	F: CTGGAAGTGGAATCCGTTTG R: CGACCTCAGCGAAATAGTTG	JF411604
GPX1a^5^	F: GTGACGACTCTGTGTCCTTG R: AACCTTCTGCTGTATTCTCTTGA	JF411605
GPX1b^6^	F: TATGTCCGTCCTGGCAATGG R: ATCGCTGGGAATGGAAGTT	JF411606
occludin	F:ATCGGTTCAGTACAATCAGG R:GACAATGAAGCCCATAACAA	KF975606
ZO-1^7^	F:GCCTGCCTACACTCAACCACAAC R:CTGCTTCGGCTGGAGGAGGAG	KY290394.1
Claudin3	F:GCACCAACTGTATCGAGGATG R:GGTTGTAGAAGTCCCGAATGG	JQ767157
Claudin7	F:CTTCTATAACCCCTTCACACCAG R:ACATGCCTCCACCCATTATG	JQ767155
Claudin11	F:TCGGAAGTGAACCAGAAAGC R:GAAGCCAAAGGACATCAAGC	JQ767158
MLCK^8^	F:AGCAGTGTGGGCATCAACCT R:CTCCAGCAGGGTCATGATGAG	XM_019076433.1
IL-1β^9^	F:AACTTCACACTTGAGGAT R: GACAGAACAATAACAACAAC	KC008576
IL-6α^10^	F: TAGGTTAATGAGCAAGAGGA R: AGAGACTGTTGATACTGGAA	AY102633.1
IL-8^11^	F:AAACTGAGAGTCGACGCATTG R:TTTTCAATGACCTTCTTAACCCAG	EU011243.1
IL-10^12^	F:GCCAGCATAAAGAACTCG R:CCAAATACTGCTCGATGT	JX524550.1
TNF-α^13^	F:AAGTCTCAGAACAATCAGGAA R: TGCCTTGGAAGTGACATT	AJ311800
TNF-β2^14^	F: GGGACATCATCGCCATCT R: TGACATTCTCGGCAGGGT	U66874.1
β-actin	F:GATCGGCAATGAGCGTTTCC R: ACGGTGTTGGCATACAGGTC	M24113.1

1. Nfr2: nuclear factor (erythroid-derived 2)-like 2; 2. Keap1: Kelch-like ECH-associated protein 1; 3. CuMnSOD: CuMn-superoxide dismutase; 4. CAT: catalase; 5. GPX1a: glutathione peroxidase 1a; 6. GPX1b: glutathione peroxidase 1b; 7.ZO-1: Zonula occluden 1; 8. MLCK: Myosin Light Chain Kinase; 9. IL-1β: Interleukin-1β; 10. IL-6α: Interleukin-6; 11. IL-8: Interleukin-8; 12. IL-10: Interleukin-10; 13. TNFα: Tumor Necrosis Factorα 14. TNF β2: Tumor Necrosis Factor β2; And F stands for Forward; R stands for Reverse.

### Analysis of Gut Microbiota

The intestinal contents of the abovementioned three fish from the same aquarium were collected, mixed into one, frozen with liquid nitrogen, and stored at −80°C for later analyses of intestinal microflora communities after 7 h of feeding, when their feeding digestion was almost accomplished and the feces had reached the hindgut. The intestinal content samples were transported to Shanghai Majorbio Bio-pharm Technology Co., Ltd., immediately under a low-temperature environment of solid carbon dioxide (dry ice). With respect to DNA extraction, library preparation, and sequencing, detailed information was specifically stated (5). Quantitative Insights into Microbial Ecology (QIIME, v1.9.0) software was used for quality control splicing, filtering, and other pre-processing of the raw data.

### Data Processing and Analysis


Survival rate (SR; %) = 100 × final number (fish)/initial number (fish)

Feed intake (FI; g/fish) = total feed intake per cage (g)/(number of fish ∗82 days)

Weight gain rate (WGR;%) = 100 × [final weight(g) −initial weight (g)] / final weight (g)

Feed conversion rate(FCR)= 100 × feed supplied (g)/weight gain (g)

Specific growth rate(SGR; %/day)= 100 × [(ln final weight − ln initial weight)/ 82days]

$$\begin{array}{l} \text{Protein efficiency ratio (PER;\%) = 100\% ×[final weight }\left(\text{g}\right)\;-\text{ initial weight }\left(\text{g}\right)\text{)/(total feed intake }\left(\text{g}\right)\text{ × }\\ \text{content of dietary protein }\left(\%\right)] \end{array}$$

All data are represented as means ± SD. Results were analyzed using SPSS 22.0 software (SPSS Inc., Chicago, IL, USA) by methods of two-way ANOVA and Duncan’s test to delineate significance among the groups. For all analyzes, *p* < 0.05 was chosen as a significant level unless otherwise stated.

## Results and Analysis

### Growth Performance and Feed Utilization

Growth performance was significantly affected by carbonate alkalinity and dietary phosphorus levels (*p* < 0.05). Irrespective of carbonate alkalinity, the higher final body weight, WGR, SGR, and PER at the 7.0 g/kg phosphorus group and the lower feed conversion ratio (FCR) were observed as compared to those in the 3.6 and 10.5 g/kg groups (*p* < 0.05). Moreover, despite dietary phosphorus levels, fish fed at carbonate alkalinity of 15 mmol/L gained lower WGR, SGR, PER, and FI and higher FCR as compared to those fed at carbonate alkalinity of 0 mmol/L (*p* < 0.05). There was no significant interaction of carbonate alkalinity and dietary phosphorus level on growth performance and feed utilization (*p* > 0.05) (**Table 5**).

**Table 5:** Growth performance and feed utilization of Songpu mirror carp at different treatments.

Carbonate alkalinity levels (mmol/L)	Phosphoruslevels (g/kg)	Initial body weight/g	Final body weight/g	Weight gain rate (WGR, %)	Specific growth rate (SGR, %/d)	Protein efficiency ratio (PER, %)	Feed conversion ratio (FCR)	Feed intake (FI, g/fish)
0	3.6	2.94±0.09	12.46±1.41^b^	322.61±15.13^b^	1.75±0.04^b^	1.43±0.04^b^	2.33±0.06^b^	0.40±0.02^b^
	7.0	3.02±0.11	16.16±0.85^a^	438.18±5.61^a^	2.05±0.01^a^	1.63±0.02^a^	2.04±0.03^a^	0.49±0.01^a^
	10.5	3.21±0.14	14.66±1.12^ab^	357.45±35.43^b^	1.84±0.10^b^	1.51±0.11^ab^	2.21±0.10^ab^	0.45±0.03^ab^
15	3.6	2.77±0.06	9.08±0.13^d^	227.77±4.41^c^	1.45±0.02^c^	1.14±0.02^c^	2.93±0.08^c^	0.33±0.01^c^
	7.0	2.82±0.27	12.46±1.9^b^	340.03±15.02^b^	1.80±0.04^b^	1.41±0.06^b^	2.37±0.11^b^	0.41±0.02^b^
	10.5	2.94±0.23	9.59±0.37^c^	226.98±10.84^c^	1.44±0.04^a^	1.18±0.02^c^	2.84±0.04^c^	0.34±0.01^c^
Carbonate alkalinity	0	3.05	14.46^p^	372.75^p^	1.88^p^	1.52^p^	2.19^p^	0.44^p^
	15	2.85	10.38^q^	264.92^q^	1.56^q^	1.24^q^	2.72^q^	0.36^q^
Phosphorus levels	3.6	2.86	10.76^y^	275.19^x^	1.60^y^	1.28^y^	2.63^x^	0.36^y^
	7.0	2.92	14.36^x^	389.11^x^	1.92^x^	1.52^x^	2.21^y^	0.45^x^
	10.5	3.06	12.13^y^	275.19^y^	1.64^y^	1.35^y^	2.53^x^	0.39^y^
*P*-value of two-way ANOVA								
Phosphorus levels		0.13	0.00	0.00	0.00	0.00	0.00	0.00
Carbonate alkalinity		0.07	0.00	0.00	0.00	0.00	0.00	0.00
Carbonate alkalinity * Phosphorus levels		0.89	0.51	0.42	0.36	0.42	0.09	0.45

all the values are expressed as the mean values±SD (n=6), and different letters in the same column denote a significant difference (p<0.05). *means the interaction between carbonate alkalinity and phosphorus levels.

### Antioxidant Capacity in the Intestine

As shown in **Table 6**, there was a similar tendency for the increasing carbonate alkalinity to decrease the glutathione peroxidase (GSHPx) activities in the intestine. Ignoring the effect of dietary phosphorus level, fish reared at carbonate alkalinity of 15 mmol/L obtained lower superoxide dismutase (SOD) and GSHPx activities compared to those reared at carbonate alkalinity of 0 mmol/L, only with no significant difference between the two carbonate alkalinity treatments (*p* > 0.05). Additionally, compared to the intestine with the 0 mmol/L carbonate alkalinity treatment, the intestine with the 15 mmol/L carbonate alkalinity treatment had higher MDA contents, albeit with no significant difference between the two carbonate alkalinity treatments (*p* > 0.05).

**Table 6:** Antioxidant capacity in the intestine of Songpu mirror carp at different treatments.

Carbonate alkalinity levels (mmol/L)	Phosphoruslevels (g/kg)	Superoxide dismutase(SOD, U/mgprot)	Glutathione peroxidase(GSHPx, U/gprot)	Malondialdehyde(MDA, nmol/mgprot)
0	3.6	182.41±3.77^ab^	86.16±13.79^b^	17.02±1.86^b^
	7.0	186.01±2.82^ab^	457.39±77.49^a^	9.66±0.54^c^
	10.5	213.72±1.24^a^	353.11±758.27^a^	11.71±0.55^bc^
15	3.6	158.21±22.61^b^	113.03±23.92^b^	18.49±0.08^a^
	7.0	180.67±19.63^ab^	367.70±63.70^a^	10.13±1.27^bc^
	10.5	202.60±15.77^a^	121.45±7.51^b^	13.12±0.21^b^
Carbonate alkalinity	0	194.05	298.89^p^	12.79
	15	180.49	200.73^q^	13.91
Phosphorus levels	3.6	170.31^y^	99.60^z^	17.76^y^
	7.0	183.34^xy^	412.55^x^	9.90^y^
	10.5	208.16^x^	237.28^y^	12.42^x^
Phosphorus levels		0.03	0.03	0.00
Carbonate alkalinity		0.20	0.02	0.19
Carbonate alkalinity * Phosphorus levels		0.74	0.74	0.85

all the values are expressed as the mean values±SD (n=6), and different letters in the same column denote a significant difference (p<0.05). *means the interaction between carbonate alkalinity and phosphorus levels.

SOD and GSHPx activities and malondialdehyde (MDA) contents were significantly affected by dietary phosphorus levels (*p* < 0.05). SOD activities in the intestine of fish maintained at the same carbonate alkalinity treatment significantly rose with the increase in dietary phosphorus levels from 3.6 to 10.5 g/kg (*p* < 0.05). For fish maintained at the same carbonate alkalinity treatment, significantly higher GSHPx activities and lower MDA contents were observed in the 7.0 g/kg phosphorus treatment (*p* < 0.05).

### Relative Expression Levels of Antioxidant Genes in the Intestine

The relative expression levels of antioxidant genes in the intestine are presented in **Table 7**. A significant effect of carbonate alkalinity was merely found in the relative expression levels of Nfr2 and GPX1a in the intestine. Regardless of dietary phosphorus level, carbonate alkalinity of 15 mmol/L can significantly decrease the relative expression levels of Nfr2 and GPX1a in the intestine, compared with carbonate alkalinity of 0 mmol/L (*p* < 0.05). Otherwise, there was a tendency for carbonate alkalinity of 15 mmol/L to downregulate the relative expression levels of CuZnSOD, CAT, and GPX1b, although this downregulation was not significant between the two carbonate alkalinity treatments (*p* > 0.05). In contrast, there was a tendency for carbonate alkalinity of 15 mmol/L to upregulate the relative expression levels of Keap1, although no significant difference was observed (*p* > 0.05).

**Table 7:** Relative expression levels of antioxidant genes in the intestine of Songpu mirror carp at different treatments.

Carbonate alkalinity levels (mmol/L)	Phosphoruslevels (g/kg)	Nfr2	Keap1	CuZnSOD	CAT	GPX1a	GPX1b
0	3.6	0.92±0.06^ab^	1.49±0.71	1.72±0.38^ab^	0.88±0.08^ab^	1.68±0.38^ab^	1.70±0.41^ab^
	7.0	1.65±0.08^a^	0.90±0.20	2.25±0.59^a^	1.51±0.47^a^	1.90±0.27^a^	2.08±0.45^a^
	10.5	0.89±0.11^ab^	1.02±0.27	1.66±0.50^ab^	0.87±0.11^ab^	2.33±0.74^ab^	2.02±0.07^ab^
15	3.6	0.86±0.01^ab^	2.07±0.89	0.70±0.05^b^	0.66±0.14^b^	0.99±0.13^b^	1.45±0.68^b^
	7.0	0.96±0.08^ab^	1.88±0.70	1.88±0.40^ab^	1.01±0.32^ab^	1.25±0.27^ab^	1.66±0.23^ab^
	10.5	0.72±0.03^b^	0.94±0.24	1.47±0.07^ab^	0.76±0.08^ab^	1.26±0.32^ab^	1.21±0.29^ab^
Carbonate alkalinity	0	1.16^p^	1.14	1.88	1.09	1.97^p^	1.93
	15	0.85^q^	1.63	1.34	0.81	1.17^q^	1.44
Phosphorus levels	3.6	0.89	1.78	1.21	0.77	1.34	1.58
	7.0	1.31	1.39	2.06	1.27	1.58	1.87
	10.5	0.80	0.98	1.56	0.82	1.80	1.62
Phosphorus levels		0.15	0.40	0.13	0.13	0.53	0.74
Carbonate alkalinity		0.03	0.31	0.12	0.19	0.03	0.16
Carbonate alkalinity * Phosphorus levels		0.24	0.65	0.55	0.73	0.85	0.78

all the values are expressed as the mean values±SD (n=6), and different letters in the same column denote a significant difference (p<0.05). *means the interaction between carbonate alkalinity and phosphorus levels.

From **Table 8**, it was shown that except for Keap1, the relative expression levels of other antioxidant genes in the intestine reached their maximum values when the fish were fed the 7.0 g/kg phosphorus diets for fish maintained at the same carbonate alkalinity treatment, although there was no statistically significant difference among three phosphorus treatments (*p* > 0.05). Additionally, the relative expression levels of Keap1 tended to elevate with increasing dietary phosphorus levels despite carbonate alkalinity (*p* > 0.05).

**Table 8:** Relative expression levels of tight junction complexes (TJs) in the intestine of Songpu mirror carp at different treatments.

Carbonate alkalinity levels (mmol/L)	Phosphoruslevels (g/kg)	occludin	zonula occludins 1(ZO-1)	claudin1	claudin2	claudin3	claudin7	claudin11
0	3.6	0.86±0.21^ab^	1.88±0.70	1.73±0.37^ab^	0.88±0.08^ab^	1.68±0.38	1.70±0.41	1.38±0.20^b^
	7.0	1.66±0.48^a^	2.06±0.89	2.25±0.59^a^	1.51±0.47^a^	1.90±0.48	2.08±0.45	2.23±0.07^b^
	10.5	0.72±0.06^b^	0.93±0.24	1.66±0.50^ab^	0.87±0.19^ab^	2.33±0.73	2.02±0.07	3.50±0.24^a^
15	3.6	0.92±0.32^ab^	0.90±0.70	0.69±0.05^b^	0.66±0.14^b^	0.99±0.13	1.45±0.68	0.67±0.08^c^
	7.0	0.96±0.13^ab^	1.49±0.13	1.88±0.40^ab^	1.01±0.32^ab^	1.25±0.27	1.66±0.23	2.27±0.28^b^
	10.5	0.88±0.19^ab^	1.02±0.27	1.47±0.07^ab^	0.76±0.08^ab^	1.27±0.32	1.21±0.29	0.33±0.10^c^
Carbonate alkalinity	0	1.08	1.62	1.87	1.09	1.97^p^	1.93	2.37^p^
	15	0.93	1.14	1.34	0.81	1.17^q^	1.44	1.09^q^
Phosphorus levels	3.6	0.89	1.39	1.21	0.77	1.34	1.57	1.02^y^
	7.0	1.31	1.78	2.06	1.27	1.58	1.87	2.25^x^
	10.5	0.80	0.98	1.56	0.81	1.80	1.62	1.92^xy^
*P*-value of two-way ANOVA								
Phosphorus levels		0.15	0.40	0.13	0.13	0.53	0.74	0.00
Carbonate alkalinity		0.47	0.31	0.12	0.20	0.03	0.16	0.00
Carbonate alkalinity * Phosphorus levels		0.22	0.65	0.54	0.73	0.85	0.78	0.00

all the values are expressed as the mean values±SD (n=6), and different letters in the same column denote a significant difference (p<0.05). *means the interaction between carbonate alkalinity and phosphorus levels.

### Relative Expression Levels of Tight Junction Complexes in the Intestine

The relative expression levels of TJs in the intestine are assumed in **Table 8**. **Table 8** shows that the relative expressions of claudin3 and claudin11 in the intestine were merely significantly influenced by carbonate alkalinity levels (*p* < 0.05). The claudin3 and clautin11 expression levels were markedly downregulated by increasing carbonate alkalinity levels up to 15 mmol/L (*p* < 0.05). Also, the slight downregulations of the expression levels of occludin, zonula occludens 1 (ZO-1), claudin1, claudin2, and claudin7 in the intestine were observed when carbonate alkalinity was elevated up to 15 mmol/L, although there was no apparent difference with the 0 mmol/L carbonate alkalinity treatment (*p* > 0.05).

There was no remarkable effect of dietary phosphorus level on relative expression levels of TJs (*p* > 0.05). Irrespective of the carbonate alkalinity levels, their maximum values were detected in the 7.0 g/kg phosphorus treatment, but no obvious changes were observed in all groups (*p* > 0.05). Here, it was notable that the interaction of dietary phosphorus levels and carbonate alkalinity levels was observed in the relative expression of claudin11 (*p* < 0.05).

### Relative Expression Levels of Cytokines in the Intestine

The relative expression levels of cytokines in the intestine are shown in **Table 9**. As pro-inflammatory cytokines, the interleukin 1β (IL-1β), IL-6α, IL-8, and TNF-α expression levels in the intestine were upregulated by increasing carbonate alkalinity levels up to 15 mmol/L. Among these, there was a significant increment in the TNF-α expression levels at the 15 mmol/L carbonate alkalinity treatment compared to the 0 mmol/L carbonate alkalinity treatment (*p* < 0.05). On the contrary, as anti-inflammatory cytokines, fish maintained at carbonate alkalinity of 15 mmol/L obtained the markedly lower TNF-β and MyD88, which belongs to anti-inflammatory cytokines, in comparison to those maintained at carbonate alkalinity of 0 mmol/L (*p* < 0.05).

**Table 9:** Relative expression levels of cytokines in the intestine of Songpu mirror carp at different treatments.

Carbonate alkalinity levels (mmol/L)	Phosphoruslevels (g/kg)	IL-1β	IL-6α	IL-8	IL-10	TNF-α	TNF-β	MyD88
0	3.6	2.67±1.99^b^	5.40±0.13^ab^	1.83±0.02	4.62±1.82	0.94±0.23^b^	1.27±0.38^ab^	0.95±0.11^a^
	7.0	1.47±0.27^b^	4.30±0.69^b^	1.21±0.50	5.67±1.01	0.85±0.40^b^	1.75±0.43^a^	1.01±0.25^a^
	10.5	2.52±0.31^b^	4.81±0.38^ab^	1.01±0.07	4.00±0.82	0.79±0.19^b^	0.65±0.09^b^	0.67±0.12^ab^
15	3.6	3.90±1.49^ab^	7.61±1.27^a^	1.91±0.41	2.49±0.64	1.94±0.15^a^	0.61±0.16^b^	0.44±0.14^b^
	7.0	7.04±0.18^a^	4.08±1.54^b^	1.89±0.45	4.53±1.31	1.70±0.49^ab^	0.86±0.21^b^	0.66±0.10^ab^
	10.5	7.21±0.24^a^	6.88±0.95^ab^	1.39±0.13	1.90±0.58	1.68±0.07^ab^	0.58±0.12^b^	0.36±0.03^b^
Carbonate alkalinity	0	2.22^q^	4.83	1.30	4.76	0.86^q^	1.22^p^	0.88^p^
	15	6.05^p^	6.19	1.78	2.97	1.78^p^	0.68^q^	0.48^q^
Phosphorus levels	3.6	3.29	6.50^x^	1.87	3.56	1.44	0.94^y^	0.70^xy^
	7.0	4.26	4.19^y^	1.55	5.09	1.28	1.31^x^	0.84^x^
	10.5	4.87	5.84^xy^	1.21	2.94	1.24	0.62^y^	0.52^y^
*P*-value of two-way ANOVA								
Phosphorus levels		0.13	0.04	0.16	0.18	0.77	0.04	0.04
Carbonate alkalinity		0.02	0.11	0.24	0.07	0.00	0.03	0.00
Carbonate alkalinity * Phosphorus levels		0.54	0.40	0.51	0.88	0.97	0.32	0.76

all the values are expressed as the mean values±SD (n=6), and different letters in the same column denote a significant difference (p<0.05). *means the interaction between carbonate alkalinity and phosphorus levels.

**Table 10** shows that the relative expression levels of IL-6α, TNF-β, and MyD88 in the intestine were markedly affected by dietary phosphorus levels (*p* < 0.05). Ignoring the carbonate alkalinity, the maximum levels of TNF-β and MyD88 were observed in the 7.0 g/kg phosphorus treatment, and the minimum levels of IL-6α and TNF-α were also observed in the 7.0 g/kg phosphorus treatment.

**Table 10:** Relative abundance of Fusobacteria, Bacteroidetes, Proteobacteria, Firmicutes, and ratio of Firmicutes/Bacteroidetes in the intestine of Songpu mirror carp under different conditions.

Items	*Fusobacteria*	*Bacteroidetes*	*Proteobacteria*	*Firmicutes*	*Firmicutes/Bacteroidetes*
**For fish fed with diets containing different phosphorus levels at 0 carbonate alkalinity group**					
LP_C0	0.04±0.01^b^	0.20±0.08	0.43±0.07^a^	0.09±0.03^a^	0.83±0.62
NP_C0	0.80±0.13^a^	0.03±0.02	0.11±0.09b	0.01±0.00^b^	1.09±0.47
HP_C0	0.78±0.08^a^	0.03±0.01	0.03±0.00^b^	0.02±0.01^b^	1.22±0.83
**For fish fed with diets containing 7.0 g/kg phosphorus at 0 and 15 mmol/L carbonate alkalinity groups**					
N0_C0	0.80±0.13^a^	0.03±0.02	0.11±0.09^b^	0.01±0.00	1.09±0.47^a^
N0_C15	0.29±0.18^b^	0.06±0.04	0.47±0.13^a^	0.03±0.01	0.78±0.44^b^

all the values are expressed as the mean values±SD (n=6), and different letters in the same column denote a significant difference (p<0.05).

### Intestinal Microflora

Based on the analysis of the above results, we preliminarily judged that 7.0 g/kg phosphorus addition was more appropriate for Songpu mirror carp reared either at the 0 mmol/L carbonate alkalinity group or at the 15 mmol/L carbonate alkalinity group. Hence, in the determination of intestinal microflora, we mainly analyzed the effects of different phosphorus levels on the intestinal microflora of fish at the 0 mmol/L carbonate alkalinity group (LP_C0, NP_C0, and HP_C0 groups), and the effects of 7.0 g/kg phosphorus addition on the intestinal microflora of fish at the 0 mmol/L carbonate alkalinity group (NP_C0 group) or 15 mmol/L carbonate alkalinity group (NP_C15 group).

### Microbiota of the Intestine Richness and Diversity Analysis

**Figure 1:**
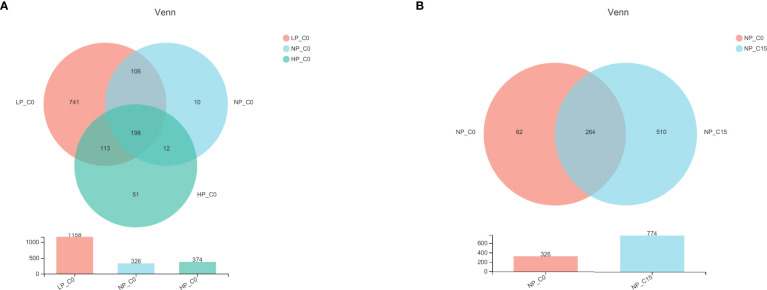
Venn diagram demonstrating the distribution of operational taxonomic units (OTUs) shared by Songpu mirror carp fed with different phosphorus levels at 0 mmol/L carbonate alkalinity group **(A)** and Songpu mirror carp fed with diets containing 7.0 g/kg phosphorus addition at 0 mmol/L carbonate alkalinity group or 15 mmol/L carbonate alkalinity group **(B)**. Note: LP_C0, 3.6 g/kg of phosphorus and 0 mmol/L carbonate alkalinity; NP_C0, 7.0 g/kg of phosphorus and 0 mmol/L carbonate alkalinity; HP_C0, 10.5 g/kg of phosphorus and 0 mmol/L carbonate alkalinity; NP_C15, 10.5 g/kg of phosphorus and 15 mmol/L carbonate alkalinity. All the values are expressed as the mean values ± SD (n = 3).

In freshwater, a total of 198 operational taxonomic units (OTUs) were shared by 3 phosphorus treatments, and the number of unique OTUs in fish fed a diet containing the 3.6 g/kg of phosphorus was the highest (**Figure 1A**). Additionally, 15 mmol/L carbonate alkalinity obviously elevated the number of unique OTUs in fish fed with diets containing 7.0 g/kg of phosphorus, and simultaneously a total of 264 OTUs were shared by 2 carbonate alkalinity treatments (**Figure 1B**).

### Taxonomic Composition Performance Analysis and Comparison of Intestinal Microbiota

**Figure 2:**
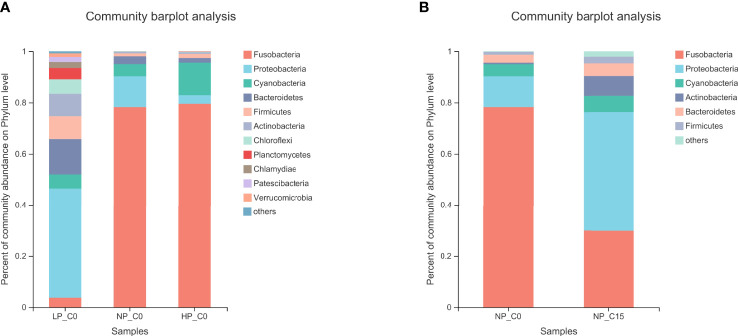
The bacterial composition of the different communities at the phylum level in Songpu mirror carp fed with different phosphorus levels at 0 mmol/L carbonate alkalinity group **(A)** and Songpu mirror carp fed with diets containing 7.0 g/kg phosphorus addition at 0 mmol/L carbonate alkalinity group or 15 mmol/L carbonate alkalinity group **(B)**. all the values are expressed as the mean values ± SD (n = 3).

As exhibited in **Figure 2**, the composition of the intestinal microbiota at the phylum level has obviously changed under different conditions. In **Figure 2A**, fish fed with diets containing 7.0 and 10.5 g/kg of phosphorus at the 0 mmol/L carbonate alkalinity group (NP_C0 and HP_C0 groups) at the phylum level was mainly composed of Fusobacteria, Bacteroidetes, Proteobacteria, and Cyanobacteria. Contrary to the NP_C0 and HP_C0 groups, a more complex diversiform composition of intestinal microbiota in fish fed with diets containing 3.6 g/kg of phosphorus at the 0 mmol/L carbonate alkalinity group (LP_C0 group) was observed. For fish fed with diets containing 7.0 g/kg of phosphorus, increasing carbonate alkalinity from 0 (NP_C0 group) to 15 mmol/L (NP_C15 group) markedly elevated the abundance of Proteobacteria, Cyanobacteria, and Bacteroidetes and decreased the abundance of Fusobacteria.

As shown in **Table 10**, Fusobacteria was the most abundant bacterium apart from the LP_C0 and NP_C15. In the 0 carbonate alkalinity group, Songpu mirror carp in the LP_C0 group obtained a significantly lower relative abundance of Fusobacteria and a higher relative abundance of Proteobacteria and Firmicutes compared to the other two groups (*p* < 0.05). Meanwhile, a decrease in the ratio of Firmicutes/Bacteroidetes was observed with increasing dietary phosphorus levels, albeit with no significant difference (*p* > 0.05). For fish fed with diets containing 7.0 g/kg phosphorus at the 0 and 15 mmol/L groups, the Fusobacteria abundance and the ratio of Firmicutes/Bacteroidetes significantly decreased with increasing carbonate alkalinity levels (*p* < 0.05), whereas the Proteobacteria abundance increased with significant differences being observed between two carbonate alkalinity levels (*p* > 0.05).

**Figure 3:**
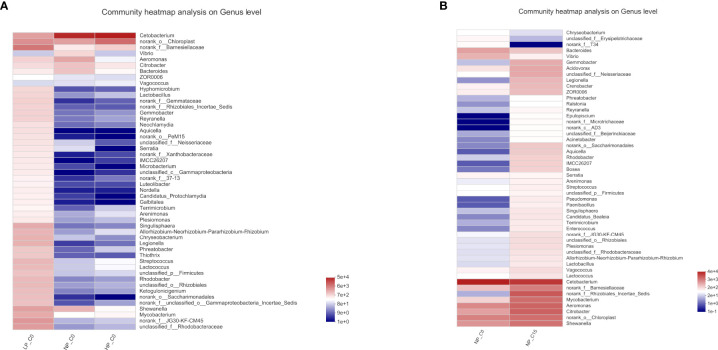
The heatmaps of the specimens show the relative abundances of the main identified bacteria at the genus taxonomic level. Red indicates a higher relative abundance, whereas blue indicates a lower relative abundance. Note: all the values are expressed as the mean values ± SD (n = 3).

The community heatmap of the relative abundances of the top 50 most abundant bacteria on the genus level was analyzed in **Figure 3**. At 0 mmol/L carbonate alkalinity, increasing phosphorus levels from 3.6 (LP_C0 group) to 10.5 g/kg (NP_C15 group) contributed to the synchronously decreasing the relative abundances of *Bacteroides*, *Hyphomicrobium*, *Lactobacillus*, *Thiothrix*, *Shewanella*, *Mycobacterium*, and *Rhodobacter* (**Figure 3A**). For fish fed with diets containing 7.0 g/kg of phosphorus, increasing carbonate alkalinity from 0 (NP_C0 group) to 15 mmol/L (NP_C15 group) markedly elevated the abundance of *Shewanella*, *Citrobacter*, *Aeromonas*, and *Mycobacterium* and decreased the abundance of *Bacteroides* (**Figure 3B**).

**Figure 4:**
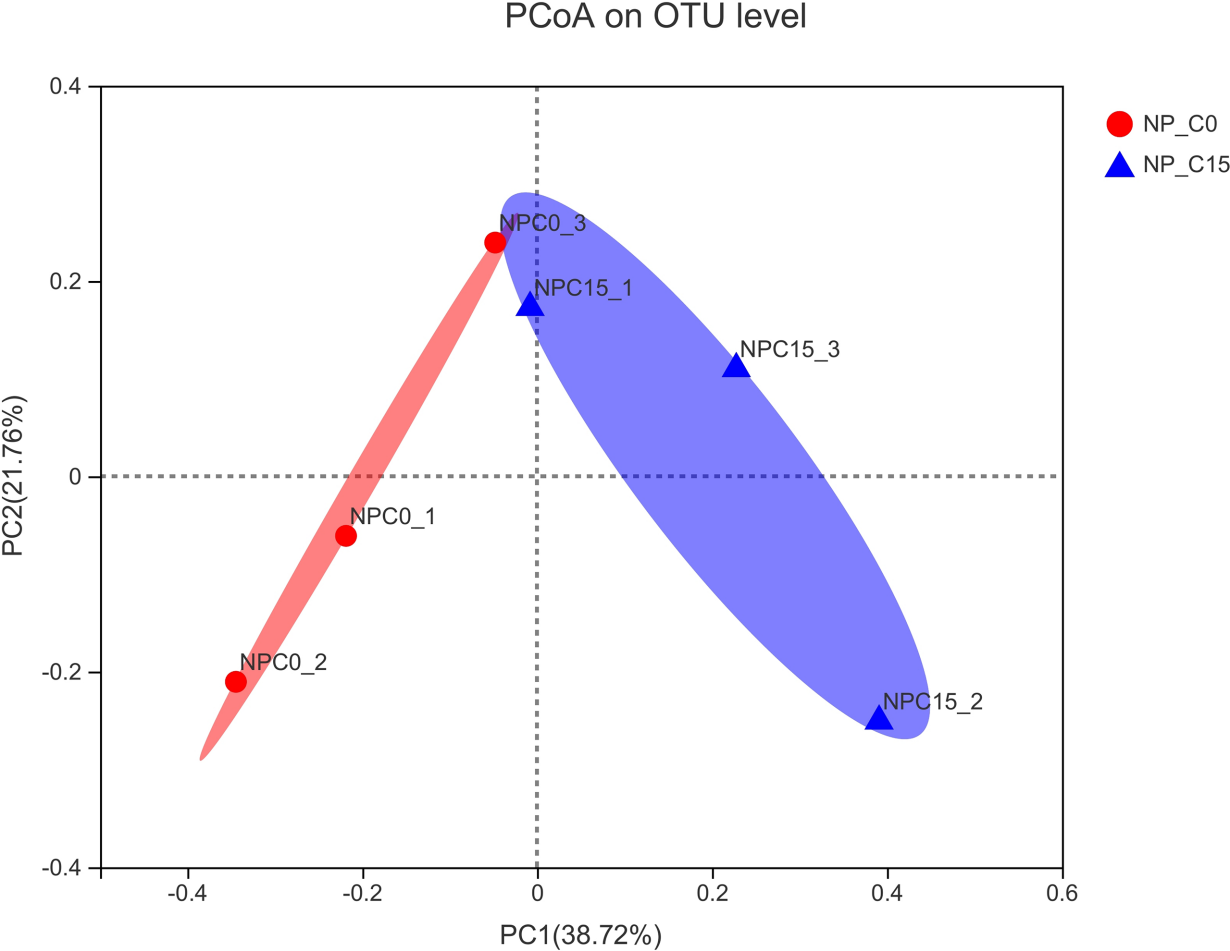
Principal coordinate analysis (PCoA) of the unweighted UniFrac scores of the microbial communities under the different conditions. Note: all the values are expressed as the mean values ± SD (n = 3).

In **Figure 4**, the overall structural changes of the intestinal microbiota were induced by increasing carbonate alkalinity levels relying on principal coordinate analysis (PCoA) whereby there was a significant structural difference regarding the beta diversity between NP_C0 and NP_C15 groups.

## Discussion

Increasing the carbonate alkalinity resulted in a decrease in WGR, SGR, and PER and an increase in FCR. This is similar to previous research whereby a significant decline in the feed ration was found after rearing *Allogynogemetic crucian* at carbonate alkalinity of 10 mmol/L (24). In this study, our purpose was to explore the effect of dietary phosphorus levels on intestinal immunity, physical barrier function, and intestinal microflora in answer to the carbonate alkalinity. Moreover, these indicators would be ultimately reflected in the growth performance. We found that, either in the 0 mmol/L carbonate alkalinity treatment or in the 15 mmol/L carbonate alkalinity treatment, the most excellent growth performance of fish was observed in the 7.0 g/kg phosphorus treatment, which also suggested that Songpu mirror carp fed with diets containing deficient or excessive phosphorus presented the depressed growth, a similar finding to gibel carp (*Carassius auratus gibelio* var. CAS III)) (25), triploid Atlantic salmon (*Salmo salar* L.) (26), triploid rainbow trout (*Oncorhynchus mykiss*) (27), nile tilapia (*Oreochromis niloticus*) (28), and grass carp (*Ctenopharyngodon idella*) (8). Meanwhile, there was no remarkable interaction between carbonate alkalinity and dietary phosphorus level on the growth indices and FCR. The above findings consistently indicated that the excessive dietary phosphorus supply was not required to alleviate the carbonate alkalinity stress. This nutrient requirement status was also observed in our previous study, which found that more than 310 g/kg of dietary protein supply can have an adverse effect on growth performance (5). Similar inference can be drawn from the results of a biochemical evaluation of digestion ability, intestinal immunity, physical barrier function, and intestinal microflora in response to the carbonate alkalinity.

Previous work on Japanese sea bass (*Lateolabrax japonicus*) has shown that their digestive enzyme activities significantly decreased after rearing them at carbonate alkalinity of 10 mmol/L (29). Similarly, we found that there was a markedly negative effect of carbonate alkalinity on the activities of lipase and amylase. These results have borne out claims that high-concentration carbonate alkalinity could become a known risk for the function of intestine digestion and absorption (4, 30). Its underlying causes are poly-factorial but the most important of these, perhaps, is that carbonate alkalinity could indirectly lead to a dramatic activity decline in digestive enzymes by means of changing the environmental factors in the intestine, especially the pH (31). In terms of diets, we found that different from the variation tendency of first increase and then decrease of lipase activities at the 0 mmol/L carbonate alkalinity treatment, increasing dietary phosphorus supply from 3.6 to 10.5 g/kg tended to be associated with a significantly increased occurrence of the lipase activities at the 15 mmol/L carbonate alkalinity treatment. However, a previous study in gibel carp (25) indicated that the lipase activities in the intestine were significantly and negatively interrelated with the increasing dietary phosphorus levels in freshwater. Concurrently, increasing dietary phosphorus supply at the 15 mmol/L carbonate alkalinity treatment tended to be associated with an increased occurrence of the amylase activities to a certain extent, although there was no notable effect. The explanation regarding these findings was presumably supposed that high dietary phosphorus could strengthen the utilization of energy substance observed in Songpu mirror carp reared at high-concentration carbonate alkalinity, indicating that dietary phosphorus may be an effective factor for mitigating the digestive problems caused by carbonate alkalinity.

Apart from the digestive function, the intestine is also a crucial immunologic barrier, including anti-oxidation capability, physical barrier function, and intestinal microflora (32). Similar to our previous study on Songpu mirror carp (4, 5), fish in this study were diagnosed with the presence of antioxidant dysfunction during the high-concentration carbonate alkalinity stress, involving reductions in the activities of SOD, CAT, and GSHPx and improvements in the contents of MDA of fish. As can be seen from the angle of molecular biology, the results in this study demonstrate that the abnormal expressions of related genes in the Keap1-Nrf2 pathway could be the deep-seated reason causing antioxidant dysfunction. In this pathway, Keap1 is a repressor protein that accelerates the cytoplasmic degradation of Nrf2, whereas Nrf2 is the key signaling protein that enhances downstream antioxidant genes transcription (33, 34). We found that the upregulated Keap1 and downregulated Nrf2 expression levels resulted in the decreased expression of downstream antioxidant enzyme genes, including CuZnSOD, CAT, GPX1a, and GPX1b, thereby failing to deal with the increased MDA or oxidative stress at the 15 mmol/L carbonate alkalinity treatment. The anti-oxidation capability and Keap1-Nrf2-ARE pathway not only can make a responsive change due to the complexity of the surrounding environment (35) but also can be effectively regulated by feed nutrition (36). Indeed, our previous study revealed that a dietary protein supply of 310 g/kg or 1% addition of AKG in diets could effectively alleviate the oxidative stress induced by carbonate alkalinity (4, 5). Current research has shown that the appropriate dietary phosphorus level significantly increased the activities of SOD and GSHPx in the intestine, thereby promoting fish antioxidant capacity by removing superoxide anion and lipid peroxidative product, a similar finding to Jian carp (21). Moreover, it was noted that 7.0 g/kg phosphorus diets at two carbonate alkalinity treatments tend to be associated with a maximum occurrence of the expression of related antioxidant genes, but no significant difference was observed, which indicated a moderate response to milder antioxidant stress generated by carbonate alkalinity. Hence further work is required in seeking an effective nutrition substance that transfers the antioxidant stress.

Under normal circumstances, epithelial TJ of the intestine is formed as transmembrane (such as occludin and members of the claudin superfamily) and cytosolic (such as ZO-1) and acts in crucial roles in the physical barrier (37). However, once the environmental stress appears, subsequent oxidative damage elicits the aberrant TJ protein expression and impaired TJ structure and function (38–40). Similar to our previous study (4, 5), 15 mmol/L carbonate alkalinity led to a decrease in TJ-related gene expressions (including occludin, ZO-1, claudin1, claudin2, and claudin7) of the intestine and especially a significant reduction in claudin3 and claudin11 expressions, which showed that superfluous carbonate alkalinity disrupted the intestinal TJ integration of Songpu mirror carp. In the field of dietary phosphorus, an early study on mice by Suzuki and Hara (41) established the fact that phosphorus can reduce calcium ion concentration in the neurons, and a low concentration of calcium ion inhibits the myosin light-chain kinase (MLCK) signaling pathway, thereby enhancing TJ protein expression. Moreover, previous work in grass carp has shown that the downregulated MLCK mRNA levels by dietary phosphorus increased the expression levels of zonula occludens, occludin, claudin-c, claudin-f, claudin7, and claudin11 and decreased claudin12, indicating that phosphorus could maintain the intercellular structural integrity in the intestine of fish (42). Similarly, we observed that at the 0 and 15 mmol/L carbonate alkalinity treatments, the intestine of Songpu mirror carp in the 7.0 g/kg phosphorus treatment obtained higher expression levels of TJ-related gene, and especially significantly higher claudin11 expression level, which suggested that consideration should be given to suitable phosphorus supply for fish reared at the high-concentration carbonate alkalinity environment. This hypothesis also could be testified by evidence that remarkable interaction between carbonate alkalinity and dietary phosphorus occurs in the claudin11 expression level. Previous research by John et al. (43) established the truth that the decreased expression of claudin11, which participates in the formation of a TJ barrier, can reduce the barrier function in human intestinal epithelial cells. This could explain the underlying principle that dietary phosphorus generates an antagonistic effect on carbonate alkalinity stress for fish.

In addition to impaired epithelial TJ function of the intestine, oxidative damage induced by environmental stress may act as a trigger for high intestinal inflammation and immune response in fish (44). Generally, inflammation and immunity response would be regulated through the dynamic equilibrium between the pro-inflammatory cytokines (such as TNF-α, IL-1β, IL-6, and IL-8) and anti-inflammatory cytokines (such as IL-10 and TGF-β2) (45–47). Previous studies about individual exposure to Cu^2+^ (39) and Pb^2+^ (40) suggested that their toxic effects were manifested by the abnormal gene expression of inflammatory cytokines. In our research too, Songpu mirror carp showed upregulated pro-inflammatory cytokine (TNF-α, IL-1β, IL-6α, and IL-8) and anti-inflammatory cytokine expression (IL-10 and TGF-β2) during the chronic stress of carbonate alkalinity. This indicated that acute intestinal inflammation has arisen, accompanying impaired epithelial TJ function, which further led to the aberrant intestinal structure and function (48). Here, it should be noted that nutrient substances in diets such as protein (36), phospholipids (49), pantothenic acid (50), and manganese (Mn) (51) could participate in modulating the intestinal inflammation and immune response of fish. Likewise, we found that in the appropriate dietary phosphorus (7.0 g/kg) treatment, acute intestinal inflammation induced by carbonate alkalinity tended to be relieved *via* both enhancing the expressions of anti-inflammatory factors (IL-10 and TNF-β) and weakening the expressions of pro-inflammatory factors (IL-6α, IL-8, and TNF-α) in the intestines. As Chen et al. (42) reported, optimal phosphorus level upregulated the gene expressions of TOR and S6K1 and downregulated 4E-BP, causing the upregulation of anti-inflammatory cytokines IL-4/13A, IL-10, IL-11, TGF-β1, and TGF-β2 gene expressions, indicating that phosphorus inhibited the inflammation in the mucosal immune and body immune organs of fish, which was in agreement with our findings. Thus, according to the above results, the 7.0 g/kg phosphorus supplementation could be beneficial for the intestinal health of Songpu mirror carp cultured in the high-concentration carbonate alkalinity environment.

In view of the fact that the intestinal microflora of fish plays a part in a great deal of functions, involving growth, host nutrient absorption, immunity, and disease state (52), acquiring an in-depth understanding of the interplay between the breeding environment (53) or feeding conditions (54) and intestinal microflora is crucial with regard to fish health. Unlike mammalian species, next-generation sequencing (NGS) results revealed that microorganisms in the intestine of fish mainly consist of Proteobacteria, Fusobacteria, Firmicutes, Bacteroidetes, Actinobacteria, and Verrucomicrobia. Among these, Proteobacteria, Bacteroidetes, Actinobacteria, Firmicutes, and Fusobacteria are considered the dominant bacterial communities at the phylum level (55, 56), which is in line with our current findings regardless of carbonate alkalinity and dietary phosphorus level. When considering the carbonate alkalinity in this study, the results of PCoA diversity analysis revealed that chronic carbonate alkalinity stress could change the structural composition at the phylum level of the intestinal microflora in fish fed with diets containing 7.0 g/kg of phosphorus. However, what should not be ignored is that dietary phosphorus supply also leads to the remarkable variation of intestinal microflora at the 0 mmol/L carbonate alkalinity treatment, although the results of the PCoA diversity analysis are remarkable. By comparing the results of intestinal microflora under different conditions, significant increments in the abundance of Proteobacteria and a significant decrease in the abundance of Fusobacteria and the ratio of Firmicutes/Bacteroidetes were caused by excessively high carbonate alkalinity (15 mmol/L) and excessively low dietary phosphorus supply (3.6 g/kg). Generally, Proteobacteria occurs an abnormal surplus in certain conditions, such as hypoxia stress for cobia (*Rachycentron canadum*) (57), ammonia-N stress for Chinese mitten crab (*Eriocheir sinensis*) (58), excessively high salinity for yellow drum (*Nibea albiflora*) (59), excessive dietary protein for Songpu mirror carp (5), and excessive dietary fish oil for rainbow trout (*O. mykiss*) (55), thereby resulting in inflammatory intestinal disease. Hence, the variation tendency of the Proteobacteria abundance should need to be focused on when environmental conditions and feed nutrition have been sharply changed. Here, in terms of intestinal inflammatory status, we found that the abundance of *Shewanella*, which belong to the pathogenic bacterium, was also observed to markedly increase by comparing the results of intestinal microflora at the genus taxonomic level. It suggested that a potentially pathogenic environment in the intestine of fish would be established by chronic carbonate alkalinity stress or deficiency of dietary phosphorus (60). However, to our amazement, the more species and quantity of pathogenic bacterium in this study were not observed in comparison to our previous study that assessed the interaction of carbonate alkalinity and dietary protein on intestinal microflora, which reported that the relative abundance of three kinds of pathogenic bacterium, including *Shewanella*, *Vibrio*, and *Flavobacterium*, increased with increasing carbonate alkalinity (5). The Firmicutes/Bacteroidetes (F/B) ratio has been identified as an important factor in regulating the growth and energy metabolism (57, 61, 62), which indicated that carbonate alkalinity stress or deficiency of dietary phosphorus restrains the energy metabolism to impair the growth of fish. Based on these results, the fact should be recognized that an appropriate dietary phosphorus supply could promote the formation of beneficial microflora in freshwater, and it has a potential ability to transfer the adverse effect of carbonate alkalinity stress to the structural composition of intestinal microflora. In the current study, the underlying mechanism of Proteobacteria and F/B ratio and a certain pathogenic bacterium is undiscovered, and further exploration should be henceforth required in a future study.

## Conclusions

**Figure 5:**
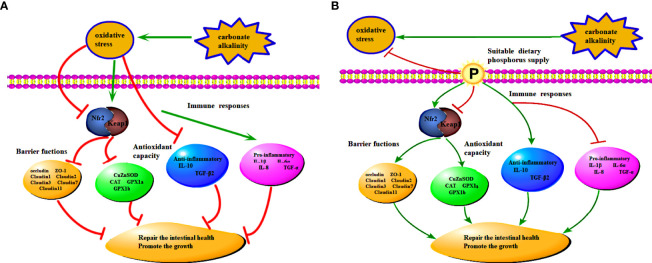
**(A)** The mechanism of carbonate alkalinity stress induces impaired growth and intestinal health. **(B)** The underlying mechanism of dietary phosphorus supply transfers the adverse effect of carbonate alkalinity stress to growth and intestinal health.

When reared in the high-concentration carbonate alkalinity environment, Songpu mirror carp suffer from hypoimmunity status with failed digestion, antioxidant, inflammation, and immune response, thereby inducing impaired growth (**Figure 5A**). Collectively, as summarized in **Figure 5B**, the current study provided the first experimental evidence that 7.0 g/kg dietary phosphorus supplementation was effective in promoting intestinal antioxidant enzyme activities and the corresponding gene expression *via* the Keap1-Nrf2 signaling pathway and in enhancing intestinal immunity by upregulating anti-inflammatory and downregulating pro-inflammatory genes, which then improve the growth performance of Songpu mirror carp at the 15 mmol/L carbonate alkalinity environment. Furthermore, the intestinal microflora in Songpu mirror carp was found to be altered in response to carbonate alkalinity. Appropriate dietary phosphorus supply could promote the formation of beneficial microflora in freshwater, and it has the potential ability to transfer the adverse effect of carbonate alkalinity stress to the structural composition of intestinal microflora. On all accounts, consideration should be given to suitable phosphorus supply for fish under the chronic carbonate alkalinity stress.

## Data Availability Statement

All data generated or used during the study appear in the submitted article, without undue reservation. This data can be found here: https://www.ncbi.nlm.nih.gov/bioproject/PRJNA844243.

## Ethics Statement

All Animal procedures in this study were conducted according to the guidelines for the care and use of laboratory animals of Heilongjiang River Fisheries Research Institute (CAFS). The studies in animals were reviewed and approved by the Committee for the Welfare and Ethics of Laboratory Animals of Heilongjiang River Fisheries Research Institute.

## Author Contributions

LW and XZ contributed to the conceptualization and investigation. ZF contributed to the project administration, writing of the original draft preparation, formal analysis, and resources. DW contributed to the formal analysis and investigation. JL and CL contributed to the formal analysis. All authors contributed to the article and approved the submitted version.

## Funding

This work was supported by the Central Public-interest Scientific Institution Basal Research Fund, CAFS (2022XT0402), the Central Public-interest Scientific Institution Basal Research Fund, HRFRI (HSY202111Q, HSY202002M), the National Natural Science Foundation of China (31802305), the China Agriculture Research System of MOF and MARA.

## Conflict of Interest

The authors declare that the research was conducted in the absence of any commercial or financial relationships that could be construed as a potential conflict of interest.

## Publisher’s Note

All claims expressed in this article are solely those of the authors and do not necessarily represent those of their affiliated organizations, or those of the publisher, the editors and the reviewers. Any product that may be evaluated in this article, or claim that may be made by its manufacturer, is not guaranteed or endorsed by the publisher.

## References

[1] Liu YX Fang H Lai QF Liang LQ. The Current State and Development Strategy for China's Saline-Alkaline Fisheries. Strat Stud CAE (2016) 18:74–8.

[2] Sun YC Wu S Du NN Song Y Xu W. High-Throughput Metabolomics Enables Metabolite Biomarkers and Metabolic Mechanism Discovery of Fish in Response to Alkalinity Stress. RSC Adv (2018) 8:14983–90. doi: 10.1039/c8ra01317a PMC9079986 35541358

[3] Tu HQ Zhao JL Huang SY Hao YY Hao YM Cao XY. Ammonia Transporter Expression of Rh Protein Gene in Gills of Nile Tilapia *Oreochromis Niloticus* Under Stress of Alkali. Fishe Sci (2019) 38:194–200. doi: 10.16378/j.cnki.1003-1111.2019.02.007

[4] Fan Z Peng F Li JN Wu D Zhang YY Wang CA. Effects of α-Ketoglutarate on Growth Performance, Antioxidant Capacity and Ammonia Metabolization Against Chronic Carbonate Alkalinity Stress in Songpu Mirror Carp (*Cyprinus Carpio* Songpu). Aqua Res (2020) 51:2029–40. doi: 10.1111/are.14554

[5] Fan Z Wu D Zhang YY Li JY Xu QY Wang LS. Carbonate Alkalinity and Dietary Protein Levels Affected Growth Performance, Intestinal Immune Responses and Intestinal Microflora in Songpu Mirror Carp (*Cyprinus Carpio* Songpu). Aquaculture (2021) 545:737135. doi: 10.1016/j.aquaculture.2021.737135

[6] Jobling M. National Research Council (NRC): Nutrient Requirements of Fish and Shrimp. Aquacul Int (2012) 20(3):601–2. doi: 10.1007/s10499-011-9480-6

[7] Masuda K Boyd CE. Phosphorus Fractions in Soil and Water of Aquaculture Ponds Built on Clayey Ultisols at Auburn, Alabama. J World Aquacul Soc (2007) 25(3):379–95. doi: 10.1111/j.1749-7345.1994.tb00222.x

[8] Liang JJ Liu YJ Tian LX Yang HJ Liang GY. Dietary Available Phosphorus Requirement of Juvenile Grass Carp (*Ctenopharyngodon Idell*a). Aquacult Nutr (2012) 18:181–8. doi: 10.1111/j.1365-2095.2011.00887

[9] Ai F Wang LS Li JN Xu QY. Effects of a-Ketoglutarate (AKG) Supplementation in Low Phosphorous Diets on the Growth, Phosphorus Metabolism and Skeletal Development of Juvenile Mirror Carp (*Cyprinus Carpio*). Aquaculture (2019) 507:393–401. doi: 10.1016/j.aquaculture.2019.03.047

[10] Sun Y Li B Zhang X Chen M Tang H Yu X. Dietary Available Phosphorus Requirement of Crucian Carp, Carassius Auratus. Aquacult Nutr (2018) 00:1–8. doi: 10.1111/anu.12686

[11] Xie NB Feng L Liu Y Jiang J Jiang WD Hu K. Growth, Body Composition, Intestinal Enzyme Activities and Microflora of Juvenile Jian Carp (*Cyprinus Carpio* Var. Jian) Fed Graded Levels of Dietary Phosphorus. Aquacult Nutr (2011) 17:645–56. doi: 10.1111/j.1365-2095.2011.00867.x

[12] Zafar N Khan MA. Determination of Dietary Phosphorus Requirement of Stinging Catfish *Heteropneustes Fossilis* Based on Feed Conversion, Growth, Vertebrae Phosphorus, Whole Body Phosphorus, Haematology and Antioxidant Status. Aquacult Nutr (2018) 24:1577–86. doi: 10.1111/anu.12794

[13] Fjelldal PG Hansen T Huang T. Continuous Light and Elevated Temperature can Trigger Maturation Both During and Immediately After Smoltification in Maleatlantic Salmon (*Salmo Salar*). Aquaculture (2011) 321:93–100. doi: 10.1013/j.auqaculture.2011.08.017

[14] Zhang X Liang MQ Wei YL Liao ZB Zhang QG Xu HG. Effects of Dietary Protein Content and Stocking Density on Growth Performance, Nitrogen Excretion, and Relevant Biochemical Parameters of Juvenile *Takifugu rubripes* . Progr Fish Sci (2021) 42(1):74–83. doi: 10.19663/j.issn2095-9869.20191028001

[15] Fjelldal PG Hansen T Breck O Sandvik R Waagbø R Berg A. Supplementation of Dietary Minerals During the Early Seawater Phase Increase VerTebral Strength and Reduce the Prevalence of Vertebral Deformities in Fast-Growing Under-Yearling Atlantic Salmon (*Salmo Salar* L.) Smolt. Aquacult Nutr (2009) 15:366–78. doi: 10.1111/j.1365-2095.2008.00601.x

[16] Xu ZL Zeng BH Yang RB Mou ZB Li BH Wang WL. Effects of Dietary Protein Level on Immune and Antioxidant Capacity of Juvenile *Schizopygopsis Younghusbandi* Regan. Chin J Anim Nutrit (2019) 31(12):5645–54. doi: 10.3969/j.issn.1006-267x.2019.12.030

[17] Broderick NA. A Common Origin for Immunity and Digestion. Front Immunol (2015) 6:**72**. doi: 10.3389/fimmu.2015.00072 PMC4333870 25745424

[18] Sun FL Wang YS Wang CZ Zhang L Tu K Zheng ZP. Insights Into the Intestinal Microbiota of Several Aquatic Organisms and Association With the Surrounding Environment. Aquaculture (2019) 507:196–202. doi: 10.1016/j.aquaculture.2019.04.026

[19] Hu XS Li CT Shang M Ge YL Jia ZY Wang SH. Inheritance of Growth Traits in Songpu Mirror Carp (*Cyprinus Carpio* L.) Cultured in Northeast China. Aquaculture (2017) 477:1–5. doi: 10.1016/j.aquaculture.2017.04.031

[20] National Research Council (NRC). Washington, DC: National Academy Press: Nutrient Requirements of Fish and Shrimp. (2011).

[21] Dong M Feng L Kuang SY Liu Y Jiang J Hu K. Growth, Body Composition, Intestinal Enzyme Activities and Microflora of Juvenile Jian Carp (*Cyprinus Carpio* Var. Jian) Fed Graded Levels of Dietary Valine. Aquacul Nutr (2013) 19:1–14. doi: 10.1111/j.1365-2095.2011.00926.x

[22] AOAC (Association of Official Analytical Chemists). Official Methods of Analysis of the Association of Official Analytical Chemists International. 16th edition. Arlington, Virginia, USA: AOAC (2006).

[23] Pfaffl MW. A New Mathematical Model for Relative Quantification in Real-Time RT PCR. Nucleic Acids Res (2001) 29:2002–7. doi: 10.1093/nar/29.9.e45 PMC55695 11328886

[24] Lv F Huang JT Wang AM. Effects of Alkalinity on the Food Consumption, Growth and Survival of Allogynogemetic Crucian Carp. J Anhui Agric Sci (2017) 22):6789–90. doi: 10.13989/j.cnki.0517-6611.2007.22.107

[25] Xie D Han D Zhu X Yang Y Jin J Liu H. Dietary Available Phosphorus Requirement for on-Growing Gibel Carp (*Carassius Auratus* Gibelio Var. CAS III). Aquacult Nutr (2017) 23:1104–12. doi: 10.1111/anu.12478

[26] Fjelldal PG Hansen TJ Lock EJ Wargelius A Fraser TWK Sambraus F. Increased Dietary Phosphorous Prevents Vertebral Deformities in Triploid Atlantic Salmon (*Salmo Salar* L.). Aquacult Nutr (2016) 22:72–90. doi: 10.1111/anu.12238

[27] Deschamps MH Poirier Stewart N Demanche A Vandenberg GW. Preliminary Study for Phenotypic Description of Vertebral Abnormalities in Triploidtrout Subjected to Prolonged Deficiency in Phosphorus. J Appl Ichthyol (2014) 30:833–9. doi: 10.1111/jai.12518

[28] Schamber CR Boscolo WR Natali MRM Michelato M Furuya VRB Furuya WM. Growth Performance and Bone Mineralization of Large Nile Tilapia (*Oreochromis Niloticus*) Fed Graded Levels of Available Phosphorus. Aquacult Int (2014) 22:1711–21. doi: 10.1007/s10499-014-9776-4

[29] Yuan YR. Comparisons of the Digestive Enzyme Activities Between Wild and Cultivated *Lateolabrax Japonicus* and Effects of Alkalinity on the Digestive Enzyme Activities of *Lateolabrax Japonicus* Juveniles. Qingdao: Ocean Univ China (2011) 51–2.

[30] Cheng L Peng F Li JN Xu QY Wang L. Effect of Different Carbonate Alkalinity and Dietary α-Ketoglutarate Levels on Intestinal Morphology and Activity of Digestive Enzymes of Mirror Carp. Chinese Journal of Fisheries (2020) 33(1):19–24. doi: 1005-3832(2020)01-0019-06

[31] Dang YF Xu W Geng LE Bai YY. A Review of Effects of Saline-Alkalinity and pH on Growth and Development in Fish. Chin J Fishe (2021) 25:62–4. doi: 10.3969/j.issn.1005-3832.2012.02.015

[32] Luo JB Feng L Jiang WD Liu Y Wu P Jiang J. The Impaired Intestinal Mucosal Immune System by Valine Deficiency for Young Grass Carp (*Ctenopharyngodon Idella*) is Associated With Decreasing Immune Status and Regulating Tight Junction Proteins Transcript Abundance in the Intestine. Fish Shellfish Immun (2014) 40:197–207. doi: 10.1016/j.fsi.2014.07.003 25014314

[33] Kannan MB Solovieva V Blank V. The Small MAF Transcription Factors MAFF, MAFG and MAFK: Current Knowledge and Perspectives. BBA-MOL Cell Res (2012) 1823(10):1841–6. doi: 10.1016/j.bbamcr.2012.06.012 22721719

[34] Nguyen T Nioi P Pickett CB. The Nrf2-Antioxidant Response Element Signaling Pathway and its Activation by Oxidative Stress. J Biol Chem (2009) 284:13291–5. doi: 10.1074/jbc.R900010200 PMC2679427 19182219

[35] Paritosh M Pallab S Arindam B Arpan DB Anindita C Muthammal S. Mixture Effect of Arsenic and Fluoride at Environmentally Relevant Concentrations in Zebrafish (*Danio Rerio*) Liver: Expression Pattern of Nrf2 and Related Xenobiotic Metabolizing Enzymes. Aquat. Toxicol (2019) 213:105219. doi: 10.1016/j.aquatox.2019.06.002 31195325

[36] Xu J Wu P Jiang WD Liu Y Jiang J Kuang SY. Optimal Dietary Protein Level Improved Growth, Disease Resistance, Intestinal Immune and Physical Barrier Function of Young Grass Carp (*Ctenopharyngodon Idella*). Fish Shellfish Immun (2016) 55:64–87. doi: 10.1016/j.fsi.2016.05.021 27211261

[37] Peterson LW Artis D. Intestinal Epithelial Cells: Regulators of Barrier Function and Immune Homeostasis. Nat Rev Immunol (2014) 14(3):141–53. doi: 10.1038/nri3608 24566914

[38] Rao RK Yan F Polk D Brent Seth A. Probiotics Prevent Oxidative Stress-Induced Disruption of Intestinal Epithelial Barrier Function by Egf Receptor, Pkc and Map Kinase-Dependent Mechanism. FASEB J (2007) 21(5):A585–5. doi: 10.1096/fasebj.21.5.A585-a

[39] Meng XL Zhu ZX Li S Hu WP Ming H Nie GX. Effect of Waterborn Copper on the Intestinal Microbiota and Immunity of Common Carp (*Cyprtnus Carpio* L). J Henan Normal Univ (Nat Sci Edition) (2018) 46(3):85–90. doi: 10.16366/j.cnki.1000-2367.2018.03.014

[40] Liu HS Fu SL Qiu M Lin MH Wang AL Ye JM. The Effect Function of Lead (Pb) in Water on the Intestinal Structure and of Juvenile Grass Carp (*Ctenopharyngodon Ulellus*). J South China Normal Cniversity (Nat Sci Edition) (2019) 51(6):61–8. doi: 10.6054/j.jscnun.2019103

[41] Suzuki T Hara H. Difructose Anhydride III and Sodium Caprate Activate Paracellular Transport *via* Different Intracellular Events in Caco-2 Cells. Life Sci (2006) 79(4):401–10. doi: 10.1016/j.lfs.2006.01.044 16566947

[42] Chen K Zhou XQ Jiang WD Wu P Liu Y Jiang J. Impaired Intestinal Immune Barrier and Physical Barrier Function by Phosphorus Deficiency: Regulation of TOR, NF-κb, MLCK, JNK and Nrf2 Signalling in Grass Carp (*Ctenopharyngodon Idella*) After Infection With Aeromonas Hydrophila. Fish Shellfish Immun (2018) 74:175–89. doi: 10.1016/j.fsi.2017.12.060 29305994

[43] John ML Padfield PJ Burt JPH Catherine ON. Ochratoxin A Increases Permeability Through Tight Junctions by Removal of Specific Claudin Isoforms. Am J Physiol-Cell Ph (2004) 287(5):C1412–7. doi: 10.1152/ajpcell.00007.2004 15229101

[44] Kruidenier L Kuiper I Lamers CB Verspaget HW. Intestinal Oxidative Damage in Inflammatory Bowel Disease: Semi-Quantification, Localization, and Association With Mucosal Antioxidants. J Pathol (2003) 201(1):28–36. doi: 10.1002/path.1409 12950014

[45] Sanjabi S Zenewicz LA Kamanaka M Flavell RA. Anti-Inflammatory and Proinflammatory Roles of TGF-Beta, IL-10, and IL-22 in Immunity and Autoimmunity. Curr Opin Pharmacol (2009) 9(4):447–53. doi: 10.1016/j.coph.2009.04.008 PMC2755239 19481975

[46] Wu N Wang B Cui ZW Zhang XY Cheng YY Xu X. Integrative Transcriptomic and Micrornaomic Profiling Reveals Immune Mechanism for the Resilience to Soybean Meal Stress in Fish Gut and Liver. Front Physiol (2018) 9:1154. doi: 10.3389/fphys.2018.01154 30246797 PMC6140834

[47] Zheng J Ou WH Mai KS Zhang YJ. Research Progress of Plant Protein on Intestinal Health of Mariculture Fish. Anim Nutr (2019) 31(3):1072–80. doi: 10.3969/j.issn.1006-267x.2019.03.012

[48] Wei HC Chen P Liang XF Yu HH Wu XF Han J. Plant Protein Diet Suppressed Immune Function by Inhibiting Spiral Valve Intestinal Mucosal Barrier Integrity, Anti-Oxidation, Apoptosis, Autophagy and Proliferation Responses in Amur Sturgeon (*Acipenser Schrenckii*). Fish Shellfish Immun (2019) 94:711–22. doi: 10.1016/j.fsi.2019.09.061 31574297

[49] Chen YP Jiang WD Liu Y Jiang J Wu P Zhao J. Exogenous Phospholipids Supplementation Improves Growth and Modulates Immune Response and Physical Barrier Referring to NF-Kb, TOR, MLCK and Nrf2 Signaling Factors in the Intestine of Juvenile Grass Carp (*Ctenopharyngodon Idella*). Fish Shellfish Immun (2015) 47:4662. doi: 10.1016/j.fsi.2015.08.024 26306855

[50] Li L Feng L Jiang WD Jiang J Wu P Kuang SY. Dietary Pantothenic Acid Deficiency and Excess Depress the Growth, Intestinal Mucosal Immune and Physical Functions by Regulating NF-Kb, TOR, Nrf2 and MLCK Signaling Pathways in Grass Carp (*Ctenopharyngodon Idella*). Fish Shellfish Immun (2015) 45:399–413. doi: 10.1016/j.fsi.2015.04.030 25957886

[51] Jiang WD Tang RJ Liu Y Kuang SY Jiang J Wu P. Manganese Deficiency or Excess Caused the Depression of Intestinal Immunity, Induction of Inflammation and Dysfunction of the Intestinal Physical Barrier, as Regulated by NF-Kb, TOR and Nrf2 Signalling, in Grass Carp (*Ctenopharyngodon Idella*). Fish Shellfish Immun (2015) 46:406–16. doi: 10.1016/j.fsi.2015.06.007 26072140

[52] Sun Y Yang H Ling Z Chang J Ye J. Gut Microbiota of Fast and Slow Growing Grouper Epinephelus Coioides. Afr J Microbiol Res (2009) 3:637–40. doi: 6FE1AE814643

[53] Dehler CE Secombes CJ Martin SAM. Environmental and Physiological Factors Shape the Gut Microbiota of Atlantic Salmon Parr (*Salmo Salar* L.). Aquaculture (2017) 467:149–57. doi: 10.1016/j.aquaculture.2016.07.017 PMC5142738 28111483

[54] Xun PW Lin HZ Wang RX Huang Z Zhou CP Yu W. Effects of Dietary Vitamin B1 on Growth Performance, Intestinal Digestion and Absorption, Intestinal Microflora and Immune Response of Juvenile Golden Pompano (*Trachinotus Ovatus*). Aquaculture (2019) 506:75–83. doi: 10.1111/anu.12802

[55] Desai AR Links MG Collins SA Mansfield GS Drew MD Van Kessel AG. Effects of Plant-Based Diets on the Distal Gut Microbiome of Rainbow Trout (*Oncorhynchus Mykiss*). Aquaculture (2012) 350:134–42. doi: 10.1016/j.aquaculture.2012.04.005

[56] Ingerslev HC Strube ML von Gersdorff Jørgensen L Dalsgaard I Boye M Madsen L. Diet Type Dictates the Gut Microbiota and the Immune Response Against Yersinia Ruckeri in Rainbow Trout (*Oncorhynchus Mykiss*). Fish Shellfish Immun (2014) 40:624–33. doi: 10.1016/j.fsi.2014.08.021 25150450

[57] Wang WZ Jian-sheng Huang JS Zhang JD Wang ZL Li HJ Amenyogbe E. Effects of Hypoxia Stress on the Intestinal Microflora of Juvenile of Cobia (*Rachycentron Canadum*). Aquaculture (2021) 536:736419. doi: 10.1016/j.aquaculture.2021.736419

[58] Yang XZ Shi AY Song YM Niu C Yu XW Shi XL. The Effects of Ammonia-N Stress on Immune Parameters, Antioxidant Capacity, Digestive Function, and Intestinal Microflora of Chinese Mitten Crab, Eriocheir Sinensis, and the Protective Effect of Dietary Supplement of Melatonin. Comp Biochem Phys C (2021) 250:109127. doi: 10.1016/j.cbpc.2021.109127 34252579

[59] Tian L Tan P Yang L Zhu WL Xu DD. Effects of Salinity on the Growth, Plasma Ion Concentrations, Osmoregulation, non-Specific Immunity, and Intestinal Microbiota of the Yellow Drum (*Nibea Albiflora*). Aquaculture (2020) 528:735470. doi: 10.1016/j.aquaculture.2020.735470

[60] Han ZR Sun JF Lv AJ Sung YY Shi HY Hu XC. Isolation, Identification and Characterization of Shewanella Algae From Reared Tongue Sole, Cynoglossus Semilaevis Günther. Aquaculture (2017) 468:356–62. doi: 10.1016/j.aquaculture.2016.10.038

[61] Ming TY Yue G Yu TS Fang Y Li Z Yi Z. Short-Term Chronic Intermittent Hypobaric Hypoxia Alters Gut Microbiota Composition in Rats. Biomed Environ Sci (2018) 31(12):898–901. doi: 10.3967/bes2018.122 30636661

[62] Barlow GM Yu A Mathur R. Role of the Gut Microbiome in Obesity and Diabetes Mellitus. Nutr Clin Pract (2015) 30:787–97. doi: 10.1177/0884533615609896 26452391

